# Eccentric Hamstring Strength and Instrumented Anterior Knee Laxity in Professional Male Football Players: A Cross-Sectional Study

**DOI:** 10.3390/life16101634

**Published:** 2026-09-30

**Authors:** Georgios Kakavas, Nicola Maffulli, Vasileios Korakakis, Nikolaos Malliaropoulos, Florian Forelli

**Affiliations:** 1 Fysiotek Spine & Sports Lab, 17562 Athens, Greece; 2 Centre for Sports and Exercise Medicine, Queen Mary University of London, London E1 4DG, UK; 3 Department of Musculoskeletal Disorders, School of Medicine, Surgery and Dentistry, University of Salerno, 84084 Baronissi, Italy; 4 School of Pharmacy and Bioengineering, Keele University Faculty of Medicine, Stoke-on-Trent ST4 7QB, UK; 5 Department of Health Sciences, School of Life Sciences and Health Sciences, PhD in Physiotherapy Program, University of Nicosia, 2417 Nicosia, Cyprus; 6 Sports Clinic, Rheumatology Department, Barts Health NHS Trust, London E1 1BB, UK; 7 Haute-Ecole Arc Santé, HES-SO University of Applied Sciences and Arts Western Switzerland, 2800 Delémont, Switzerland; 8 Orthopaedic Surgery Department, Clinic of Domont, Ramsay Healthcare, OrthoLab, 95330 Domont, France; 9 SFMKS Lab, Société Française des Masseurs-Kinésithérapeutes du Sport, 93380 Pierrefitte-sur-Seine, France

**Keywords:** anterior cruciate ligament, knee joint, hamstring muscles, muscle strength, arthrometry, football

## Abstract

**Background/Objectives:** Eccentric knee-flexor capacity and passive anterior knee behavior are commonly assessed as distinct dimensions of knee function, but their coexistence in professional football players remains incompletely characterized. This study aimed to describe anatomical right-to-left differences in eccentric hamstring force and torque, characterize load-dependent anterior knee laxity, and examine within-limb relationships among GNRB-derived variables. **Methods:** This cross-sectional study included 25 male professional football players from two Super League clubs. Participants performed three maximal Nordic hamstring exercise trials using the NordBord system, followed immediately by bilateral GNRB Rotab arthrometry at 20° of knee flexion with three measurements at 134 N and three at 200 N. **Results:** We were unable to detect statistically significant right-to-left differences in maximal eccentric hamstring torque (mean difference 6.55 N·m, 95% CI −9.65 to 22.75; *p* = 0.412; d^z^ = 0.17) or force (−11.92 N, 95% CI −29.67 to 5.83; *p* = 0.179; d^z^ = −0.28). A post hoc sensitivity analysis indicated that *n* = 25 provided 80% power only for paired effects of approximately |d^z^| ≥ 0.58; smaller between-side differences remain compatible with the reported confidence intervals. Among the 24 participants with complete arthrometry data, separate within-limb analyses showed that TMAX was higher at 200 N than at 134 N in the left knee (*p* = 0.014), whereas the device-exported ACL output, SLOPE, and TMAX were higher at 200 N in the right knee (all *p* < 0.001). These separate tests do not establish a Side × Load interaction. At 200 N, GNRB-derived variables were strongly intercorrelated within each limb (r = 0.854–0.992). **Conclusions:** Anatomical right-to-left comparisons did not identify statistically significant mean differences in eccentric hamstring measures, but the small sample and absence of limb-dominance data preclude conclusions about equivalence or individual-level symmetry. Post-NHE GNRB-derived measures showed expected load dependence and substantial overlap. These findings are descriptive and do not establish a direct relationship between hamstring strength and ACL laxity or predict ACL injury.

## 1. Introduction

Anterior cruciate ligament (ACL) injury is one of the most consequential knee injuries in football because it entails prolonged time loss, substantial rehabilitation demands, incomplete return to preinjury performance, and subsequent knee morbidity. Although ACL rupture is usually traumatic, the mechanical environment in which the ligament is loaded reflects interactions among passive restraints, joint geometry, external forces, and neuromuscular control. Experimental studies have consistently shown that quadriceps and hamstring forces can modify anterior tibial translation and ACL strain, with hamstring loading generating a posteriorly directed tibial force that may oppose anterior tibial displacement under selected knee positions and loading conditions [1,2,3,4,5,6]. Passive knee laxity may also be clinically relevant: prospective studies have associated greater anterior–posterior tibial displacement or generalized joint laxity with subsequent non-contact ACL injury, although findings vary across populations and sex [7,8,9]. These observations support evaluating active muscular capacity and passive knee behavior as complementary rather than interchangeable dimensions of knee function.

Although the present study concerns professional football players without a history of ACL injury, the broader ACL literature illustrates why muscular performance and passive laxity should be treated as separate rather than interchangeable dimensions of knee function. Work spanning ACL healing pathways, post-reconstruction functional deficits, strength-focused rehabilitation, neuromuscular alterations, and concurrent strength–laxity assessment demonstrates that recovery across these domains is heterogeneous [10,11,12,13,14,15,16,17]. These postoperative observations cannot be directly extrapolated to uninjured players, but they reinforce the value of characterizing muscular and ligamentous measures independently.

Eccentric knee-flexor capacity can be quantified in a clinically feasible manner using the Nordic hamstring exercise (NHE). Instrumented Nordic devices have demonstrated good reliability for bilateral eccentric force assessment, including in professional football players [18,19,20]. Prospective studies have linked lower eccentric knee-flexor force to subsequent hamstring strain injury in some cohorts [21], but systematic review evidence indicates that preseason NHE strength alone provides limited information for predicting future injury [22]. The NHE nevertheless remains an established training stimulus and is incorporated into hamstring injury-prevention programmes [23]. Instrumented arthrometry provides complementary information about passive anterior knee behavior. The GNRB arthrometer applies standardized anterior loads while measuring anterior tibial translation and force–displacement characteristics, with reliability depending on positioning, stabilization, examiner technique, and applied load [24,25]. Criteria-based approaches to ACL rehabilitation likewise discourage interpreting isolated strength or laxity thresholds without clinical context [26].

Despite the plausible biomechanical interaction between hamstring function and ACL loading, limited evidence describes how field-based eccentric hamstring capacity and instrumented anterior knee laxity coexist in professional male football players. This distinction is important because strength screening in professional football has shown limited ability to identify individual future injury risk [27], and single screening measures should not be treated as deterministic predictors [28]. Importantly, the archived analytical dataset did not contain participant-level variables that permitted direct NordBord–GNRB strength–laxity analyses. The present study therefore does not test whether eccentric hamstring capacity is associated with passive anterior knee laxity. The primary aim was to describe anatomical right-to-left differences in eccentric hamstring force and torque and to characterize load-dependent instrumented anterior knee laxity. A secondary aim was to examine within-limb relationships among GNRB-derived variables. Given the cross-sectional design and absence of prospective injury surveillance, the study was intended to be descriptive and hypothesis-generating rather than predictive or causal.

## 2. Materials and Methods

### 2.1. Study Design, Setting, and Reporting

This was a cross-sectional observational study using a standardized, single-session bilateral testing protocol. Data collection was conducted in Athens, Greece, from October 2022 to May 2023, during the 2022–2023 competitive season, in professional male football players from two Super League clubs. All recruitment and testing occurred after formal ethics approval on 1 October 2022. All participants completed the same testing sequence during one assessment session. The manuscript was prepared in accordance with the Strengthening the Reporting of Observational Studies in Epidemiology (STROBE) recommendations for cross-sectional studies [29].

### 2.2. Ethics

The study was conducted in accordance with the Declaration of Helsinki and was approved by the University of Athens, Department of Physical Education and Sports Science Ethics Board. The application was submitted in July 2022 and the approval letter was dated 1 October 2022; no separate protocol or approval reference number was provided in the letter. Recruitment and testing were conducted from October 2022 to May 2023, and all study procedures occurred after formal ethical approval. Informed consent was obtained from all participants.

### 2.3. Participants and Study Size

The study analyzed de-identified data from a convenience sample of 25 male professional football players under contract to two Super League clubs. The club dataset grouped playing position into two broad categories: 19 offensive players (age 26.8 ± 2.9 years) and 6 defensive players (age 23.2 ± 2.6 years), with a reported age range of 19.4–30.7 years. These were pre-existing club categories rather than a study-derived classification, and they were not used as a proxy for functional limb dominance. The archived record did not preserve the exact positional mapping used to create these two broad categories. Eligibility required no history of ACL injury and no reported hamstring injury during the preceding 12 months. A more detailed archived screening record for other knee ligament or meniscal pathology, previous lower-extremity surgery, current musculoskeletal pain, or other recent lower-extremity injuries was not available. Accordingly, the revised manuscript does not use “healthy” as a formal eligibility descriptor. Players were included when they met the documented eligibility criteria and were available for club-based testing between October 2022 and May 2023.

No a priori sample-size, power, or precision calculation was performed. Study size was determined pragmatically by the number of eligible and available players in the two participating clubs during the study period. The resulting sample should therefore be interpreted as a convenience cohort intended for descriptive and hypothesis-generating analysis rather than as a population-representative sample. A post hoc sensitivity analysis was subsequently used to quantify the detectable paired effect size with the available sample; this analysis is described in Section 2.8 and does not replace prospective sample-size planning.

### 2.4. Testing Sequence and Standardization

Participants first completed a standardized bicycle warm-up at approximately 80–100 revolutions per minute. The archived protocol did not retain the exact warm-up duration or resistance used in this cohort. Nordic hamstring testing was then performed, followed immediately by instrumented anterior knee laxity assessment. The order of testing was fixed across participants and was not counterbalanced. Standardizing the warm-up, testing sequence, rest period between Nordic trials, knee position during arthrometry, applied arthrometer forces, and examiner was intended to reduce avoidable procedural variability.

### 2.5. Nordic Hamstring Strength Assessment

Following the bicycle warm-up, participants performed three maximal Nordic hamstring exercise (NHE) trials on a NordBord system (VALD Performance, Newstead, Australia). Sixty seconds of recovery were provided between maximal trials, and force data were sampled at 50 Hz. The archived materials confirm this repetition structure and recovery interval but did not preserve sufficiently detailed metadata to verify a separate familiarization procedure, arm position, detailed trunk/hip instructions, standardized verbal encouragement, or explicit trial-rejection/repetition criteria. These elements therefore cannot be reconstructed retrospectively. The archived materials also did not preserve a specific physiological rationale for the 60-s interval; it is reported as a standardized procedural feature of the club testing protocol rather than as an evidence-based optimal recovery duration.

The prespecified strength variables retained from the NordBord analytical output were maximal eccentric hamstring torque (HMT, N·m) and maximal eccentric hamstring force (HMF, N) for each anatomical limb. The archived analytical file contained these limb-level maximal outputs but not repetition-level data or metadata specifying whether the final device export represented the single highest trial, an average across trials, or another device aggregation rule; the aggregation procedure therefore cannot be reconstructed retrospectively. In standard NordBord methodology, torque is derived from measured force together with knee-to-ankle lever-arm information [18,19,20], but the exact device-software calculation settings used in this cohort were not preserved in the archived analytical record. Limb-dominance information was not retained in the available dataset; accordingly, strength results were analyzed by anatomical side rather than dominant versus non-dominant limb. Body mass and height were also unavailable, precluding normalization of force or torque to body size.

### 2.6. Instrumented Knee Laxity Assessment

Immediately after the NHE assessment, anterior knee laxity was measured bilaterally with a GNRB Rotab arthrometer (Genourob, Laval, France). Participants were positioned supine with the knee at 20° of flexion, confirmed using a goniometer, and the tested limb was aligned in neutral rotation with the patella facing anteriorly. The leg was supported in the adjustable device cradle, the patellar support was aligned to stabilize the femur–patella complex, and the displacement sensor was positioned over the anterior tibial tuberosity, consistent with published GNRB procedures [24,25]. Participants were instructed to remain relaxed throughout loading, and patellar stabilization and limb positioning were kept as consistent as possible across repeated measurements. A single examiner performed all arthrometry measurements to limit inter-examiner variability, and the same procedure was applied to both knees. The archived record did not retain which limb was tested first, the exact patellar stabilization force used for each participant, any separate inter-measurement rest interval, device-calibration or preliminary-loading details, or explicit trial-rejection criteria beyond retention of valid device outputs.

For each knee, six sequential measurements were obtained using the same force sequence: three measurements at 134 N followed by three measurements at 200 N. The repeated observations at each load were used to generate the summary values retained in the archived analytical dataset. The exact aggregation rule was not preserved and therefore cannot be reconstructed retrospectively. These loads were selected because 134 N is an established reference load in instrumented anterior knee arthrometry and both 134 N and 200 N have demonstrated acceptable GNRB reliability; the 200-N condition characterizes the force–displacement response under a higher applied anterior force [24,25]. GNRB-derived variables were retained according to the device-export labels: ACL represented a translation-related anterior tibial displacement/ACL laxity output (mm); SLOPE represented the slope of the force–displacement curve (µm/N); and TMAX represented maximum anterior tibial translation reached during the loading ramp (mm). ACL and TMAX are translation-related outputs derived from the same force–displacement response and were not treated as independent biological constructs. Neither the archived device-export documentation nor the manufacturer documentation available for this revision provided sufficient information to reconstruct or verify the precise computational distinction between the archived ACL and TMAX fields. The outputs were treated as post-NHE, load-dependent arthrometric measures rather than as independent resting/baseline measurements or as pre- and post-exercise measurements.

### 2.7. Data Completeness, Bias, and Reproducibility

Potential measurement variability was addressed through a fixed testing sequence, standardized NHE repetition structure and inter-trial recovery, standardized knee position during arthrometry, fixed applied arthrometer forces, and use of a single examiner for GNRB testing. Limb dominance, anthropometric characteristics, detailed training exposure, and repetition-level device data were not retained in the available dataset, so analyses were restricted to variables that could be verified. All 25 participants contributed to the NordBord analysis. One participant (4% of the cohort) did not have a complete arthrometry dataset and was therefore excluded from all GNRB analyses, leaving 24 complete cases. For this participant, the archived information did not allow us to determine whether missingness involved one knee, both knees, or specific load-level measurements, and the reason for missingness was not preserved. No imputation was performed. A sensitivity analysis including this participant was not feasible because the missing raw observations were unavailable. Excluding this participant could introduce selection bias if the missingness was related to knee characteristics; the missing-data mechanism cannot be evaluated from the archived information.

### 2.8. Statistical Analysis

Continuous variables were summarized as mean ± standard deviation (SD). Distributional assumptions were assessed using Kolmogorov–Smirnov and Shapiro–Wilk tests, with Levene’s test used when homogeneity of variance was relevant. Right-to-left differences in HMT and HMF were evaluated using two-sided paired-samples *t* tests. Mean paired differences are reported with 95% confidence intervals (CIs). Cohen’s d^z^, defined as the mean paired difference divided by the standard deviation of the paired differences, was used for the paired effect size and is reported with a 95% CI. A post hoc sensitivity analysis was performed for the paired comparisons using *n* = 25 and two-sided α = 0.05 to estimate the minimum detectable |d^z^| corresponding to 80% power. This retrospective sensitivity analysis does not substitute for an a priori sample-size calculation.

The paired strength comparisons and load-dependent arthrometry comparisons corresponded to the primary descriptive aims, whereas the correlation analyses were secondary and exploratory. GNRB-derived variables were compared separately within each limb between the 134-N and 200-N loading conditions using one-factor repeated-measures analyses of variance (ANOVAs), with partial eta squared (ηp^2^) reported as the effect-size measure from the archived repeated-measures output. With two repeated load levels and 24 complete participants, the effect and error degrees of freedom were 1 and 23, respectively. Because participant-level raw data were unavailable for the present revision, a two-factor Side × Load repeated-measures model and its interaction could not be reconstructed; therefore, statistical significance in one limb and not the other is not interpreted as evidence of a between-limb difference in load response. Pearson correlation coefficients (r) were used to examine associations among GNRB-derived variables at 200 N and among their archived percentage-change variables between 134 N and 200 N. Correlation coefficients are reported with Fisher z-transformed 95% CIs. Raw two-sided *p* values are retained for transparency. To address multiplicity, a Holm step-down sensitivity analysis was additionally applied within four analysis families: the two paired strength comparisons, the six GNRB load comparisons, the six correlations at 200 N, and the six percentage-change correlations. For archived *p* values reported only as <0.001, 0.001 was used conservatively for the adjustment, so adjusted values for these tests are reported as upper bounds. A two-sided *p* < 0.05 was considered statistically significant; percentage-change correlations were considered exploratory because change scores can be influenced by baseline values and measurement error.

## 3. Results

### 3.1. Participant Characteristics

All 25 players contributed to the hamstring force and torque dataset. The sample comprised 19 offensive players (26.8 ± 2.9 years) and 6 defensive players (23.2 ± 2.6 years), all under contract to two Super League clubs and tested in Athens, Greece, between October 2022 and May 2023. The reported age range was 19.4–30.7 years.

### 3.2. Eccentric Hamstring Force and Torque

We were unable to detect statistically significant right-to-left differences for HMT (paired t(24) = 0.83, *p* = 0.412) or HMF (paired t(24) = −1.39, *p* = 0.179) (Table 1). The mean-difference CIs included zero and the paired effect sizes were small but imprecise. Holm adjustment across the two strength comparisons did not alter interpretation (adjusted *p* = 0.412 for HMT and 0.358 for HMF). With n = 25, two-sided α = 0.05, and 80% power, the minimum detectable paired effect was approximately |d^z^| = 0.58. Thus, the nonsignificant comparisons do not establish equivalence or exclude small-to-moderate between-side differences.

### 3.3. GNRB-Derived Variables Across Applied Loads

Among the 24 participants with complete arthrometry data, the 134-N and 200-N GNRB measurements were obtained after the NHE protocol and represent two levels of applied arthrometer force, not pre- and post-intervention time points. In the separate within-limb analyses, TMAX was higher at 200 N than at 134 N in the left knee (*p* = 0.014; ηp^2^ = 0.24), whereas the device-exported ACL output and SLOPE did not differ statistically. In the right knee, the device-exported ACL output, SLOPE, and TMAX were higher at 200 N (all *p* < 0.001; ηp^2^ = 0.67–0.70) (Table 2). A Holm sensitivity analysis across the six load comparisons retained the left TMAX result (adjusted *p* = 0.042) and all three right-knee results (adjusted *p* < 0.006), while left ACL and SLOPE remained nonsignificant. These separate within-limb tests do not test a Side × Load interaction and therefore do not demonstrate that the two limbs responded differently to increasing load.

### 3.4. Correlations Among GNRB-Derived Variables

At 200 N, all three GNRB-derived variables were strongly and positively correlated within each limb. Correlations ranged from r = 0.854 to 0.880 for the left knee and from r = 0.878 to 0.992 for the right knee (Table 3). All six correlations remained statistically significant in the Holm sensitivity analysis (adjusted *p* < 0.006). The very high ACL–TMAX correlation in the right knee (r = 0.992, 95% CI 0.981–0.997) indicates substantial overlap between these translation-related outputs.

When the archived percentage-change variables between 134 N and 200 N were examined, the strongest association was between ACL and TMAX for both limbs (left: r = 0.800, 95% CI 0.586–0.910, *p* < 0.001; right: r = 0.658, 95% CI 0.347–0.839, *p* < 0.001). The left SLOPE–TMAX association was r = 0.400 with an exact two-sided *p* = 0.053. After Holm adjustment across the six percentage-change correlations, only the ACL–TMAX associations remained statistically significant (adjusted *p* < 0.006 for both limbs). These percentage-change analyses are exploratory and should be interpreted cautiously because change scores may be affected by baseline values and measurement error (Table 4).

## 4. Discussion

This study described eccentric hamstring force and torque and instrumented anterior knee laxity in a sample of male professional football players tested between October 2022 and May 2023 during the 2022–2023 competitive season. Three main observations emerged. First, group-level anatomical right-to-left comparisons did not detect statistically significant mean differences in maximal eccentric hamstring torque or force, but the study was not designed or powered to establish equivalence or individual-level symmetry. Second, several post-NHE GNRB-derived measures increased when the applied anterior force increased from 134 N to 200 N, an expected feature of a force–displacement system. Third, GNRB-derived variables were strongly intercorrelated at 200 N. Taken together, these observations support a descriptive co-characterization of muscular capacity and subsequent arthrometric knee behavior rather than a mechanistic link between the two domains; the study did not directly test whether eccentric hamstring capacity determines ACL laxity.

### 4.1. Eccentric Hamstring Capacity and Anatomical-Side Interpretation

The paired strength estimates were small, but their interpretation is constrained by precision. The post hoc sensitivity analysis showed that the available sample had 80% power only for paired effects of approximately |d^z^| ≥ 0.58, and the 95% CIs around the observed effects remained compatible with potentially meaningful differences in either direction. Accordingly, failure to detect a statistically significant mean difference should not be interpreted as equivalence or individual-level symmetry. This distinction is particularly important in football, where anatomical side is not synonymous with functional dominance: right-versus-left comparisons cannot distinguish preferred kicking from support limbs. The broad offensive-versus-defensive position labels were not used as a dominance proxy because playing position does not identify an individual player’s preferred kicking or support limb. Instrumented Nordic testing remains a reliable method for quantifying eccentric knee-flexor force [18,19,20], but absolute force and torque are influenced by body mass, technique, familiarization, and lever-arm characteristics. Because body mass and height were unavailable, strength could not be normalized to body size. The present values should therefore be viewed as group-level absolute outputs obtained under this testing protocol, not as evidence of normal bilateral symmetry or as population norms. This cautious interpretation is also consistent with evidence that isolated preseason NHE strength has limited value as a stand-alone injury-prediction tool [21,22,27,28].

### 4.2. Load-Dependent Arthrometric Behavior

The increase in GNRB outputs at 200 N should primarily be interpreted as expected load-dependent behavior of the knee–device force–displacement system rather than as a novel physiological response. The 134-N and 200-N conditions were standardized arthrometer loads obtained after the NHE protocol within the same session. They do not represent an independent resting/baseline arthrometry assessment or pre- and post-exercise time points. Both loads are established in GNRB reliability work, with 134 N serving as a commonly used reference load and 200 N providing assessment at a higher applied force [24,25]. The apparently different pattern of statistical significance between the left and right knees should not be biologically interpreted as an asymmetric response: significance in one limb and nonsignificance in the other is not a test of the difference between limbs, and no Side × Load interaction could be reconstructed from the archived aggregate data. A broader sequence of applied loads could have characterized the shape and potential non-linearity of the force–displacement response more fully, but only the 134-N and 200-N conditions were available in the archived dataset. The value of these comparisons is therefore descriptive: they characterize the magnitude and internal consistency of device outputs across two established loading conditions rather than demonstrating a side-specific mechanistic phenomenon.

### 4.3. Interrelationships Among GNRB-Derived Variables

The strong correlations among ACL, SLOPE, and TMAX indicate substantial shared information within the GNRB force–displacement response. Although the outputs describe conceptually different features—translation-related measures and curve slope—they are derived from the same loading trace and should not be assumed to provide three independent biological signals. The very high ACL–TMAX correlation in the right knee (r = 0.992) is particularly consistent with redundancy between translation-related outputs. A post hoc composite score was not created because no prespecified or validated weighting scheme was available; future studies should formally assess dimensionality and determine whether a reduced set of GNRB variables or a validated composite improves interpretability and clinical efficiency. Percentage-change correlations also warrant caution because change scores can amplify baseline dependence and measurement error; after Holm correction, only ACL–TMAX change associations remained statistically significant. Importantly, the archived dataset did not permit direct participant-level correlations between NordBord force/torque and GNRB laxity measures. Biomechanical studies show that hamstring loading can influence anterior tibial translation and ACL force under selected conditions [1,2,3,4,5,6], but those experimental observations do not establish that greater eccentric NHE capacity corresponds to lower passive arthrometric laxity in vivo.

### 4.4. Clinical Interpretation and Research Implications

From a clinical perspective, combined assessment of eccentric knee-flexor capacity and instrumented anterior knee laxity may help describe distinct dimensions of knee function, but the present data do not support using one domain as a surrogate for the other. The observed values should be regarded as sample-specific descriptive estimates rather than normative reference ranges, diagnostic thresholds, injury-risk cut-offs, or targets for return-to-sport decision making. The most useful implication of the present work is therefore methodological: strength and laxity should be interpreted as complementary measurements and retained within a broader multifactorial assessment. Future research should establish reference distributions in larger cohorts stratified by sex, competitive level, body size, and functional dominance and should directly test participant-level strength–laxity relationships. It should also evaluate Side × Load effects with appropriate repeated-measures models and determine whether these measurements add prognostic or decision-making value when linked to prospective clinical outcomes.

## 5. Limitations

This study has several important limitations related to sampling and external validity. First, the sample was small and pragmatically determined, with no a priori sample-size or precision calculation, and included only 25 male professional players from two clubs (24 complete cases for GNRB analyses). The post hoc sensitivity analysis quantifies this limited statistical sensitivity but does not correct the absence of prospective planning. The findings should therefore not be generalized directly to female athletes, amateur or recreational players, athletes from other sports, or non-athletic populations. Professional players may differ systematically from recreational athletes in strength, training exposure, and knee characteristics. Limb dominance, body mass, height, body-mass-normalized strength, playing experience, detailed training exposure, and recent match exposure were unavailable, restricting both functional interpretation and adjustment for important between-player sources of variability. In addition, the documented eligibility criteria were limited to previous ACL injury and hamstring injury within 12 months; a comprehensive archived screening record for other knee or lower-extremity conditions was not available.

Second, several measurement and protocol constraints may have influenced the observed arthrometric values. The testing sequence was fixed and non-counterbalanced: three maximal NHE trials were completed before GNRB assessment, which followed immediately. Consequently, the GNRB measurements cannot be assumed to represent an independent resting condition because preceding eccentric contractions could have affected fatigue, muscle tone, relaxation, or neuromuscular state. No pre-NHE resting arthrometry condition was available. Because the 134-N measurements systematically preceded the 200-N measurements, applied load and measurement order could not be fully disentangled, and repeated loading or mechanical preconditioning may have contributed to the observed differences. Some finer procedural metadata were also not retained, including the exact bicycle warm-up duration/resistance, detailed NHE familiarization/execution and trial-aggregation metadata, GNRB limb-testing order, inter-measurement rest interval, exact participant-specific patellar stabilization force, device-calibration/preliminary-loading details, explicit trial-rejection criteria, and the repeated-measure aggregation rule. These omissions limit full procedural replication. One participant lacked a complete arthrometry record, and the archived information did not allow determination of whether missingness involved one knee, both knees, or specific load-level measurements. The missing-data mechanism could not be evaluated and a sensitivity analysis including this participant was not possible.

Third, the analytical framework is constrained by the absence of participant-level raw data. Direct NordBord–GNRB strength–laxity correlations, individual interlimb asymmetry analyses, and a formal Side × Load interaction could not be performed retrospectively. The separate within-limb load comparisons therefore cannot establish that the limbs responded differently to increasing anterior force. Multiple inferential analyses were exploratory; Holm sensitivity analyses reduced the risk of overinterpreting isolated *p* values but do not remove the imprecision associated with a small sample. The percentage-change correlations are particularly susceptible to baseline dependence and measurement error, and the very strong intercorrelations among GNRB outputs indicate that some measures contain overlapping rather than independent information. Finally, the cross-sectional design and absence of prospective injury surveillance preclude causal inference and any conclusion regarding ACL injury prediction or prevention.

## 6. Conclusions

In this convenience sample of male professional football players, anatomical right-to-left comparisons did not detect statistically significant mean differences in eccentric hamstring force or torque; these findings do not establish equivalence or individual-level symmetry. GNRB-derived measures obtained after the NHE protocol showed expected load-dependent behavior and strong intercorrelation, indicating substantial shared information among outputs from the same force–displacement response. The observed values are best considered sample-specific descriptive estimates rather than normative reference ranges or clinical thresholds. The study does not establish a direct relationship between eccentric hamstring capacity and the subsequently acquired GNRB-derived laxity measures, nor does it support prediction of ACL injury. Larger prospective studies with anthropometric normalization, functional dominance, direct participant-level strength–laxity analyses, and appropriate Side × Load modeling are required to establish clinical relevance.

## Figures and Tables

**Table 1:** Bilateral eccentric hamstring force and torque variables (n = 25; paired-samples *t* tests).

Variable	Right Limb, Mean ± SD	Left Limb, Mean ± SD	Right–Left Mean Difference (95% CI)	*p* Value	Cohen’s d^z^ (95% CI)
HMT (N·m)	179.85 ± 30.82	173.30 ± 21.36	6.55 (−9.65 to 22.75)	0.412	0.17 (−0.23 to 0.56)
HMF (N)	323.12 ± 55.79	335.04 ± 40.80	−11.92 (−29.67 to 5.83)	0.179	−0.28 (−0.67 to 0.13)

Abbreviations: CI, confidence interval; HMT, maximal eccentric hamstring torque; HMF, maximal eccentric hamstring force; SD, standard deviation. Cohen’s d^z^ is the paired standardized mean difference (mean paired difference/SD of paired differences); 95% CIs for d^z^ were obtained from the noncentral t distribution.

**Table 2:** GNRB-derived variables at 134 N and 200 N (n = 24; separate within-limb repeated-measures ANOVAs).

Limb	Variable	134 N, Mean ± SD	200 N, Mean ± SD	*p* Value	ηp^2^
Left	ACL (mm)	5.83 ± 1.80	6.20 ± 1.80	0.133	0.09
Left	SLOPE (µm/N)	40.77 ± 10.23	42.13 ± 11.14	0.257	0.06
Left	TMAX (mm)	6.33 ± 1.62	6.88 ± 1.63	0.014	0.24
Right	ACL (mm)	4.46 ± 1.37	4.75 ± 1.41	<0.001	0.70
Right	SLOPE (µm/N)	34.58 ± 9.42	37.13 ± 10.38	<0.001	0.68
Right	TMAX (mm)	4.60 ± 1.38	4.89 ± 1.41	<0.001	0.67

Note: ACL, device-exported anterior tibial translation/ACL laxity output (mm); SLOPE, slope of the force–displacement curve (µm/N); TMAX, maximum anterior tibial translation (mm); SD, standard deviation; ηp^2^, partial eta squared. ACL and TMAX are translation-related outputs from the same force–displacement response and should not be interpreted as independent biological constructs. Repeated-measures analyses were based on 24 complete arthrometry datasets (effect df = 1; error df = 23). A Holm sensitivity analysis across the six load comparisons retained left TMAX (adjusted *p* = 0.042) and all three right-knee comparisons (adjusted *p* < 0.006).

**Table 3:** Pearson correlations among GNRB-derived variables at 200 N (n = 24; Pearson test df = 22; 95% CIs from Fisher z transformation).

Limb	Variable 1	Variable 2	r (95% CI)	*p* Value
Left	ACL	SLOPE	0.880 (0.739–0.947)	<0.001
Left	ACL	TMAX	0.854 (0.687–0.935)	<0.001
Left	SLOPE	TMAX	0.863 (0.705–0.939)	<0.001
Right	ACL	SLOPE	0.878 (0.735–0.946)	<0.001
Right	ACL	TMAX	0.992 (0.981–0.997)	<0.001
Right	SLOPE	TMAX	0.878 (0.735–0.946)	<0.001

**Table 4:** Pearson correlations among archived percentage-change GNRB variables from 134 N to 200 N (n = 24; Pearson test df = 22; 95% CIs from Fisher z transformation).

Limb	Variable 1	Variable 2	r (95% CI)	*p* Value
Left	ACL	SLOPE	0.358 (−0.053–0.665)	0.086
Left	ACL	TMAX	0.800 (0.586–0.910)	<0.001
Left	SLOPE	TMAX	0.400 (−0.004–0.692)	0.053
Right	ACL	SLOPE	0.146 (−0.274–0.519)	0.495
Right	ACL	TMAX	0.658 (0.347–0.839)	<0.001
Right	SLOPE	TMAX	0.189 (−0.232–0.550)	0.376

## Data Availability

The de-identified aggregate analytical data supporting the reported results are available from the corresponding author upon reasonable request. Participant-level raw and repetition-level device data required to conduct additional post hoc analyses were not available in the archived dataset used for the present revision.
